# Acid-Sensing Ion Channel 2a (ASIC2a) Promotes Surface Trafficking of ASIC2b via Heteromeric Assembly

**DOI:** 10.1038/srep30684

**Published:** 2016-08-01

**Authors:** Hae-Jin Kweon, Dong-Il Kim, Yeonju Bae, Jae-Yong Park, Byung-Chang Suh

**Affiliations:** 1 Department of Brain & Cognitive Sciences, DGIST, Daegu 42988, Republic of Korea; 2 School of Biosystem and Biomedical Science, College of Health Science, Korea University, Seoul 02841, Republic of Korea

## Abstract

Acid-sensing ion channels (ASICs) are proton-activated cation channels that play important roles as typical proton sensors during pathophysiological conditions and normal synaptic activities. Among the ASIC subunits, ASIC2a and ASIC2b are alternative splicing products from the same gene, *ACCN1*. It has been shown that ASIC2 isoforms have differential subcellular distribution: ASIC2a targets the cell surface by itself, while ASIC2b resides in the ER. However, the underlying mechanism for this differential subcellular localization remained to be further elucidated. By constructing ASIC2 chimeras, we found that the first transmembrane (TM1) domain and the proximal post-TM1 domain (17 amino acids) of ASIC2a are critical for membrane targeting of the proteins. We also observed that replacement of corresponding residues in ASIC2b by those of ASIC2a conferred proton-sensitivity as well as surface expression to ASIC2b. We finally confirmed that ASIC2b is delivered to the cell surface from the ER by forming heteromers with ASIC2a, and that the N-terminal region of ASIC2a is additionally required for the ASIC2a-dependent membrane targeting of ASIC2b. Together, our study supports an important role of ASIC2a in membrane targeting of ASIC2b.

Acid-sensing ion channels (ASICs) are voltage-independent, proton-gated cation channels widely expressed in the nervous system^1^,^2^,^3^. They belong to the epithelial Na^+^ channel/degenerin (ENaC/DEG) superfamily of ion channels. Extracellular acidification commonly occurs in pathological conditions such as inflammation, tissue injury, ischemic stroke, and cancer^4^,^5^,^6^. Protons have also been reported to act as a neurotransmitter in the brain, and it is well known that synaptic vesicles are acidic^7^,^8^. Therefore, detection of physiological pH changes during pathological events and normal synaptic activities is indispensable for cellular activities. ASICs play roles as typical proton sensors in the central and peripheral nervous system^9^. They are implicated in nociception, learning and memory, fear, taste transduction, and mechanosensation^10^,^11^,^12^,^13^,^14^. There are six subunits (ASIC1a, ASIC1b, ASIC2a, ASIC2b, ASIC3, and ASIC4) encoded by four genes (*ACCN2*, *ACCN1*, *ACCN3*, and *ACCN4*). *ACCN2* and *ACCN1* each produce two alternative splicing variants: ASIC1a and ASIC1b from *ACCN2*, and ASIC2a and ASIC2b from *ACCN1*. The crystal structure of chicken ASIC1 has provided insight into the structure and the function of channels^15^,^16^. These studies have revealed that three subunits assemble to form a functional homo- or heterotrimeric channel. However, among the subunits, ASIC2b and ASIC4 are not known to form functional homomeric channels. Unlike ASIC4, ASIC2b has been shown to modulate the properties of other ASICs by forming heteromeric channels^17^,^18^.

An ASIC subunit is composed of two transmembrane (TM) domains, a large extracellular loop, and short cytoplasmic N- and C- termini. ASIC2a and ASIC2b have different amino acid sequences in the N-terminus, the TM1 domain, and one-third of the extracellular loop region, while the rest of the sequences are identical. Several studies have exploited the difference in sequences of two subunits to find possible proton-binding sites present in ASIC2a, and identified five amino acids (H72, D77, E78, H109, and H180), which are absent in ASIC2b, as putative proton-binding sites^19^,^20^,^21^. They reported that single amino acid change in these five residues produced a proton-insensitive channel that trafficked normally to the cell surface like ASIC2b in Chinese hamster ovary (CHO) cells^19^,^20^. However, surface expression of these mutated channels as well as ASIC2b needs to be further studied.

In cultured hippocampal neurons, ASIC2a displays somatodendritic distribution primarily in dendrites and dendritic spines^22^. When ASIC2a was heterologously expressed in human embryonic kidney (HEK) 293 cells, it was mostly detected in the plasma membrane and other intracellular locations^23^. However, according to the previous study, ASIC2b has a reticular distribution in COS-7 cells^24^. Additionally, one research group has observed that ASIC2 proteins display a perinuclear-staining pattern in vascular smooth muscle cells (VSMCs)^25^. Quite recently, Wu *et al*. has reported that ASIC2a and ASIC2b show differential surface trafficking in NIH 3T3 cells^26^. Here, we confirmed dramatically different subcellular localization of ASIC2a and ASIC2b, and further investigated underlying mechanisms for this differential surface trafficking of ASIC2 proteins.

Delivery of ion channels and receptors to the cell surface requires efficient transport between secretory apparatus, the ER, the Golgi, and the plasma membrane^27^,^28^. From the perspective of trafficking mechanisms, surface expression of ion channels is largely dependent on discrete motifs that reside in proteins, such as ER retention or export signals. Synthesized proteins containing arginine-based ER retention signals (RXR) or physiologically misfolded and improperly assembled proteins are retained in the ER via the quality control mechanism^29^,^30^,^31^,^32^,^33^,^34^. In these cases, ER retention can be antagonized by proper heteromultimeric assembly. In addition to ER retention signals, ER export signals can be utilized to regulate the surface composition of some membrane proteins. Even though the channel proteins are properly folded and assembled, exit from the ER can require specific anterograde ER export signals^27^,^35^,^36^,^37^,^38^,^39^,^40^.

In the current study, by constructing a series of ASIC2 chimeras, we found that the TM1 and the proximal post-TM1 domain of ASIC2a are critical for surface trafficking of ASIC2 channels. Further analysis of chimeras has supported that the proximal post-TM1 domain of ASIC2a is essential for generating proton-activated currents, in accordance with the previous reports^19^,^20^,^21^. Finally, our data show that ASIC2b can be delivered to the cell surface from the ER by heteromeric assembly with ASIC2a, and that the N-terminal region of ASIC2a is additionally necessary for the ASIC2a-dependent membrane targeting of ASIC2b.

## Results

### Different subcellular localization and proton-sensitivity between ASIC2a and ASIC2b in HEK293T cells

**Figure 1:**
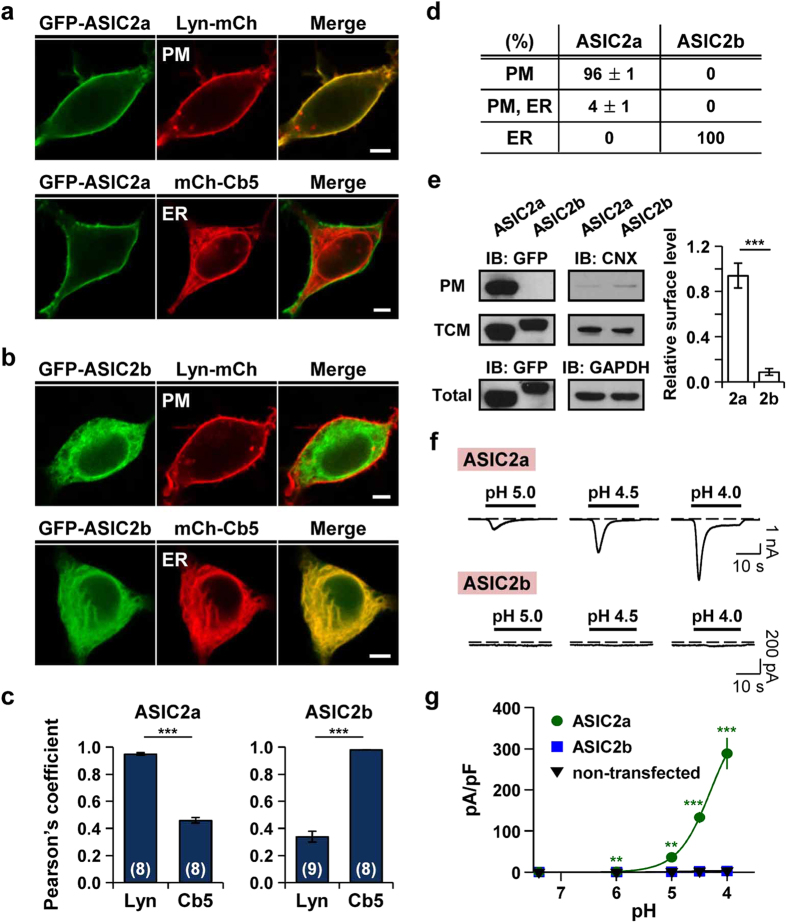
Different subcellular localization and proton-sensitivity between ASIC2a and ASIC2b in HEK293T cells. **(a,b)** Representative confocal images of HEK293T cells expressing **(a)** ASIC2a or **(b)** ASIC2b with a plasma membrane (Lyn-mCh) or ER (mCh-Cb5) probe. ASIC2a is co-localized with the plasma membrane marker, while ASIC2b is co-localized with the ER marker. The scale bar represents 5 μm. **(c)** Pearson’s correlation coefficient denoting co-localization of fluorescent images was calculated (mean ± SEM, ***p < 0.001, with Student’s two-tailed unpaired *t*-test). The number on each bar indicates n for each condition from three independent experiments. **(d)** Percentage of cells showing each subunit in specific subcellular localizations was obtained by manually counting cells co-transfected with a plasma membrane or ER probe (mean ± SEM). For each subunit, 250 cells were counted from five independent experiments. **(e)** Left, Western blotting on the plasma membrane (PM) fraction, total cellular membrane (TCM) fraction, and total lysate of cells expressing GFP-tagged ASIC2a or ASIC2b was performed using anti-GFP antibody. As controls, the PM and the TCM fractions were blotted using anti-calnexin (CNX) antibody, and total lysate was blotted using anti-GAPDH antibody. Right, the PM expression was normalized to the TCM expression (n = 5 for each, mean ± SEM, ***p < 0.001, with Student’s two-tailed unpaired *t*-test). **(f)** Proton-activated currents in HEK293T cells expressing ASIC2a (top) or ASIC2b (bottom). Rapid extracellular pH drop to indicated values from 7.4 generated the currents in cells expressing ASIC2a, while the cells expressing ASIC2b generated no currents. The time interval between pH applications is 2 min for a complete recovery from desensitization. Dashed line indicates the zero current level. **(g)** pH-dependent peak current density (mean ± SEM, **p < 0.01, ***p < 0.001, with Student’s two-tailed unpaired *t*-test compared to non-transfected). The current density of ASIC2a is increased with decreasing pH value of extracellular solution. (ASIC2a, n = 5; ASIC2b, n = 5; non-transfected, n = 6).

ASIC2a and ASIC2b differ in the N-terminus, the TM1 domain, and one-third of the extracellular loop region. For the determination of their subcellular localization in HEK293T cells, we fused GFP to N-termini of ASIC2a or ASIC2b (GFP-ASIC2a and GFP-ASIC2b, respectively), and transiently transfected into cells with a plasma membrane or ER marker. Subcellular distribution of ASIC2a and ASIC2b was examined by a confocal laser scanning microscopy 2 days after transfection. ASIC2a was predominantly localized in the cell surface, as evidenced by a yellow color in the merged image of ASIC2a and the plasma membrane marker, Lyn (N-terminal myristoylation and palmitoylation signal sequence from Lyn kinase) (Fig. 1a). A high expression of ASIC2a in the cell surface was indicated by a high value of Pearson’s correlation coefficient between ASIC2a and Lyn, compared with that measured between ASIC2a and the ER marker, Cb5 (cytochrome b5) (Fig. 1c, left). By contrast, ASIC2b was accumulated in the ER, as evidenced by the overlay image of ASIC2b and Cb5 (Fig. 1b) and a high value of Pearson’s correlation coefficient between ASIC2b and Cb5 (Fig. 1c, right). Among 250 cells transfected with ASIC2a, most cells displayed a high expression of ASIC2a in the plasma membrane 2 days after transfection (Fig. 1d). However, all of the cells transfected with ASIC2b showed its expression in the ER (Fig. 1d). We examined subcellular localization of ASIC2a and ASIC2b after different days of transfection (1 day, 2 days, and 3 days) (Supplementary Fig. S1). ASIC2a was localized in the plasma membrane already 1 day after transfection. However, ASIC2b remained in the ER even 3 days after transfection. In addition, immunostaining cells expressing HA-ASIC2a or HA-ASIC2b with anti-HA antibody revealed an identical subcellular distribution (Supplementary Fig. S1), excluding the possibility of that GFP-tagging leads to mislocalization of proteins.

Their subcellular localization was also confirmed by Western blotting. As shown in Fig. 1e, ASIC2a and ASIC2b were detected in the total cellular membrane (TCM) fraction. However, in the plasma membrane (PM) fraction, ASIC2a was strongly detected at predicted size, while ASIC2b was not. The relative surface level of ASIC2a was significantly higher than that of ASIC2b. We also performed immunoblotting on the TCM and the PM fractions with an anti-calnexin (CNX) antibody to test a purity of the PM fraction. While calnexin was strongly detected in the TCM fraction, it was barely detected in the PM fraction (Fig. 1e).

ASIC2a and ASIC2b also have differential sensitivity to extracellular protons. Extracellular acidification triggered inward currents in cells expressing ASIC2a, and the peak current density was increased with decreasing pH value of extracellular solution (n = 5) (Fig. 1f,g). However, ASIC2b generated no currents to extracellular pH drop, as previously known as a non-functional homomeric channel (n = 5) (Fig. 1f,g). There was no statistically significant difference between proton-activated currents measured from non-transfected cells (n = 6) and ASIC2b-transfected cells (Fig. 1f,g).

### Replacement of the first 129 amino acids in ASIC2b by corresponding regions of ASIC2a conferred surface expression and proton-sensitivity

**Figure 2:**
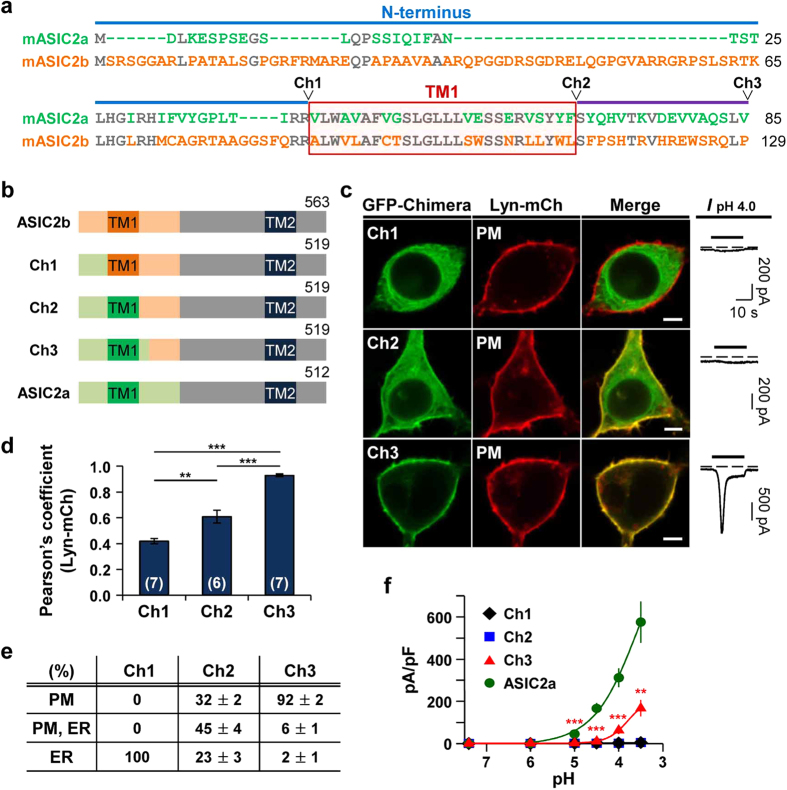
Replacement of the first 129 amino acids in ASIC2b by corresponding regions of ASIC2a conferred surface expression and proton-sensitivity. (**a**) Sequence alignment of ASIC2a (1–85 amino acids) and ASIC2b (1–129 amino acids). Blue and purple lines indicate the N-terminus and the extracellular loop region, respectively. The TM1 domain is denoted by a red box. Conserved residues between two subunits are in gray. In ASIC2b, 1–86 amino acids (aa) were replaced by 1–42 aa of ASIC2a (Ch1), 1–112 aa were replaced by 1–68 aa of ASIC2a (Ch2), and 1–129 aa were replaced by 1–85 aa of ASIC2a (Ch3). (**b**) Schematic diagram of constructed chimeras. (**c**) Left, representative confocal images of HEK293T cells expressing each chimera with the PM marker, Lyn-mCh. The scale bar represents 5 μm. Right, pH 4.0-induced currents measured from the cells expressing each chimera. Dashed line indicates the zero current level. (**d**) Pearson’s correlation coefficient between the PM marker and each chimera (mean ± SEM, **p < 0.01, ***p < 0.001, with one-way ANOVA followed by Bonferroni post-hoc test). The number on each bar indicates n for each condition from three independent experiments. (**e**) Percentage of cells showing each chimera in specific subcellular localizations (mean ± SEM). For each chimera, 250 cells were counted from five independent experiments. (**f**) pH-dependent peak current density of each channel (mean ± SEM, **p < 0.01, ***p < 0.001, with Student’s two-tailed unpaired *t*-test compared to ASIC2a). The time interval between pH applications is 2 min for a complete recovery from desensitization (Ch1, n = 5; Ch2, n = 6; Ch3, n = 5; ASIC2a, n = 5).

We created a series of chimeras of ASIC2a and ASIC2b to identify the critical regions required for membrane targeting of ASIC2a. Firstly, the N-terminus of ASIC2b was replaced by the equivalent sequences of ASIC2a (Fig. 2a,b, cf. Ch1). However, this Ch1 also resided in the ER (Fig. 2c–e), as evidenced by a high value of Pearson’s correlation coefficient between Ch1 and Cb5 (Supplementary Fig. S2). Like ASIC2b, Ch1 generated no proton-activated currents (n = 5) (Fig. 2c,f). When the TM1 domain was more replaced by that of ASIC2a (Fig. 2a,b, cf. Ch2), more than 70% of cells showed that Ch2 is expressed in the plasma membrane with partial accumulation in the ER (Fig. 2c–e). However, we could not detect any proton-activated currents in cells expressing Ch2, even though it was expressed in the cell surface (n = 6) (Fig. 2c,f). When 17 more amino acids were replaced (Fig. 2a,b, cf. Ch3), this chimera dramatically trafficked to the cell surface, as indicated by a high value of Pearson’s correlation coefficient between Ch3 and Lyn (Fig. 2c–e). Furthermore, Ch3 generated inward currents in response to protons, although the current density was significantly smaller than that of ASIC2a (n = 5) (Fig. 2c,f). Collectively, these results suggest that the TM1 and the proximal post-TM1 domain of ASIC2a are critical for membrane targeting and generating proton-activated currents.

### The TM1 and the proximal post-TM1 domain of ASIC2a are critical regions for surface trafficking of ASIC2

**Figure 3:**
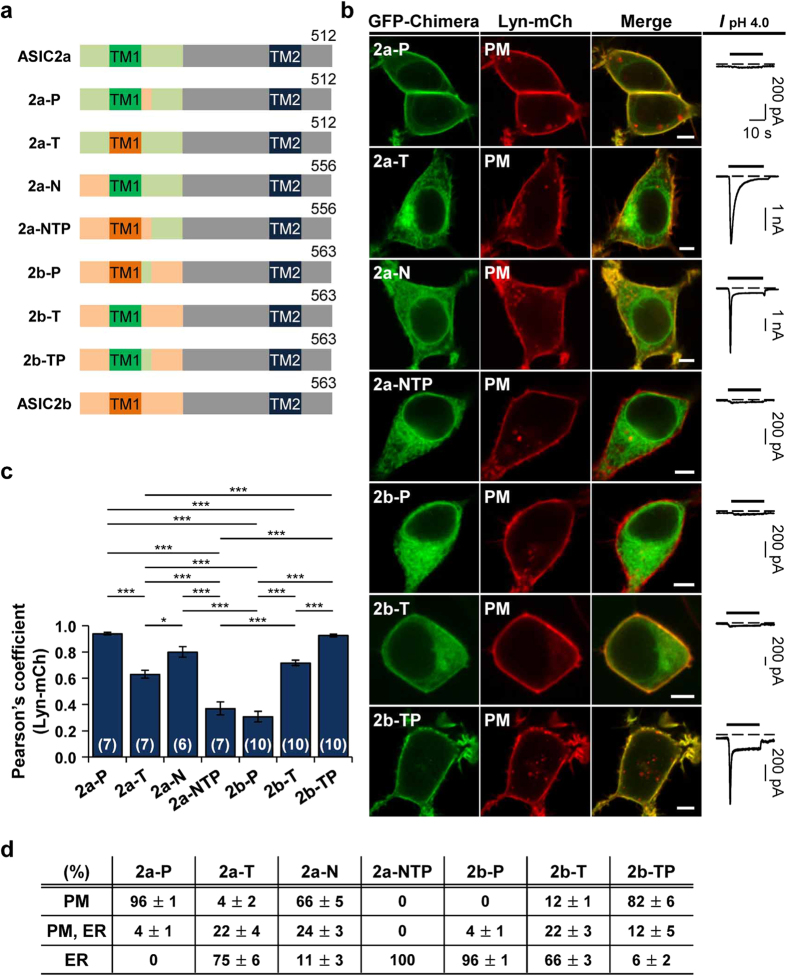
The TM1 and the proximal post-TM1 domain of ASIC2a are critical regions for surface trafficking of ASIC2. **(a)** Schematic diagram of constructed chimeras. **(b)** Left, representative confocal images of HEK293T cells expressing each chimera with the PM marker, Lyn-mCh. The scale bar represents 5 μm. Right, pH 4.0-induced currents measured from the cells expressing each chimera. Dashed line indicates the zero current level. **(c)** Pearson’s correlation coefficient between the PM marker and each chimera (mean ± SEM, * p < 0.05, ***p < 0.001, with one-way ANOVA followed by Bonferroni post-hoc test). The number on each bar indicates n for each condition from three independent experiments. **(d)** Percentage of cells showing each chimera in specific subcellular localizations (mean ± SEM). For each chimera, 250 cells were counted from five independent experiments.

Based on our observation in Fig. 2, we constructed more chimeras to verify the importance of the TM1 and the proximal post-TM1 domain in surface trafficking of ASIC2a. When the proximal post-TM1 domain in ASIC2a was replaced by that of ASIC2b (Fig. 3a, cf. 2a-P), this chimeric channel was still localized on the cell surface (Fig. 3b–d). However, it had lost proton-sensitivity (n = 5) (Fig. 3b). In the reverse chimera (Fig. 3a, cf. 2b-P), we observed that exchange of the proximal post-TM1 domain in ASIC2b to that of ASIC2a is not sufficient for exit of ASIC2b from the ER (Fig. 3b–d). This 2b-P also generated no currents in response to protons (n = 5) (Fig. 3b). However, when the TM1 domain was further replaced, this 2b-TP efficiently targeted the cell surface (Fig. 3b–d, Supplementary Fig. S3). We could also measure the proton-induced currents in cells expressing 2b-TP (n = 6) (Fig. 3b). To investigate the importance of the TM1 domain in membrane targeting, we exchanged the TM1 domain in ASIC2a with that of ASIC2b (Fig. 3a, cf. 2a-T). Interestingly, this chimeric channel displayed poor membrane localization despite retaining proton-sensitivity (n = 6) (Fig. 3b–d). Next, we examined whether the insertion of the TM1 domain of ASIC2a into ASIC2b can lead to exit of ASIC2b from the ER (Fig. 3a, cf. 2b-T). The replacement of the TM1 domain of ASIC2b by that of ASIC2a promoted forward trafficking of ASIC2b, as evidenced by a yellow color at the cell surface in the merged image of 2b-T and Lyn (Fig. 3b–d). However, 2b-T generated no currents in response to extracellular protons even at the cell surface (n = 5) (Fig. 3b). These results suggest that the TM1 domain of ASIC2a is important for surface trafficking of ASIC2, although the proximal post-TM1 domain of ASIC2a is also necessary for the efficient membrane targeting and proton-sensitivity of ASIC2.

We also tested whether the N-terminus is involved in surface trafficking of ASIC2a. When the N-terminus of ASIC2a was replaced by that of ASIC2b (Fig. 3a, cf. 2a-N), membrane localization was partially disrupted (Fig. 3b–d). When the N-terminus, the TM1, and the proximal post-TM1 domain were all replaced in ASIC2a (Fig. 3a, cf. 2a-NTP), this chimera completely lost both membrane localization and proton-sensitivity (n = 5) (Fig. 3b–d). These results suggest that orchestrated work of the TM1 domain and neighboring regions such as the N-terminus and the proximal post-TM1 domain is necessary for the efficient surface trafficking of ASIC2a. However, according to the results, replacement of the TM1 and the proximal post-TM1 domain is sufficient for conferring surface expression and proton-sensitivity on ASIC2b (Fig. 3, cf. 2b-TP).

### The N-terminus and the TM1 domain regulate the channel properties

**Figure 4:**
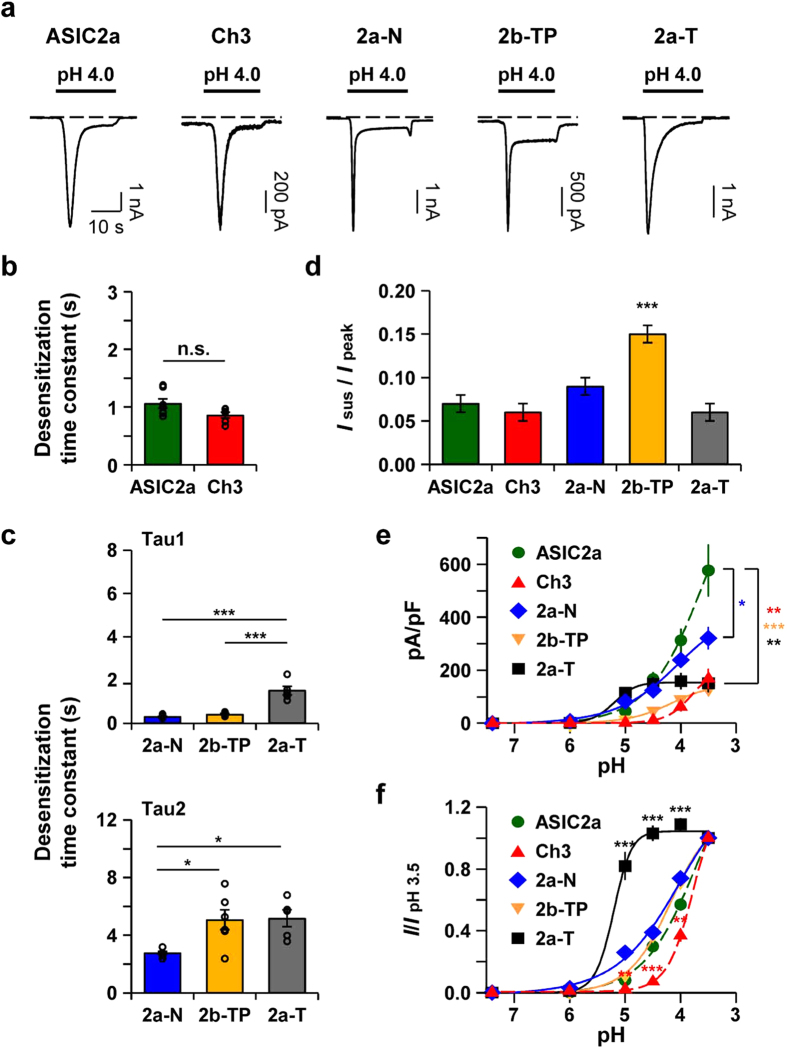
The N-terminus and the TM1 domain regulate the channel properties. **(a)** Representative current traces from cells expressing each channel. Dashed line indicates the zero current level. **(b)** Time constant (τ) for desensitization at pH 4.0 was calculated by fitting the current in a single exponential function (mean ± SEM, n.s.; not significant). **(c)** Time constants (τ) for desensitization at pH 4.0 were calculated by fitting the current in a double exponential function (mean ± SEM, * p < 0.05, ***p < 0.001, with one-way ANOVA followed by Bonferroni post-hoc test). When Area1 and Area2 represent the area of τ_1_ and τ_2_, respectively, Area1/Area2 values were 0.91 ± 0.11 (n = 6) for 2a-N, 1.40 ± 0.10 (n = 6) for 2b-TP, and 0.26 ± 0.06 (n = 5) for 2a-T. **(d)** Ratio of sustained current to peak current at pH 4.0 (mean ± SEM, ***p < 0.001, with Student’s two-tailed unpaired *t*-test compared to ASIC2a). **(e)** pH-dependent peak current density of each channel (mean ± SEM, * p < 0.05, **p < 0.01, ***p < 0.001, with Student’s two-tailed unpaired *t*-test compared to ASIC2a). The time interval between pH applications is 2 min for a complete recovery from desensitization. For 2a-N and 2b-TP, pH-dependent response was measured from individual cells. pH-dependency curves of ASIC2a and Ch3 from Fig. 2f were plotted as controls (dashed line). (ASIC2a, n = 5; Ch3, n = 5; 2a-N, n = 5 at each point; 2b-TP, n = 6 at each point; 2a-T, n = 6). **(f)** Normalized pH-dependency curves in Fig 4e. Peak current at each pH was divided by pH 3.5-evoked peak current (mean ± SEM, **p < 0.01, ***p < 0.001, with Student’s two-tailed unpaired *t*-test compared to ASIC2a).

While we performed the electrophysiological experiments with chimeras, we noticed that proton-activated currents produced by each chimeric channel have different shapes (Fig. 4a). Therefore, we further analyzed the biophysical properties of the currents by measuring the time constant (τ) for desensitization at pH 4.0. To calculate the desensitization time constant, we used a single exponential function for ASIC2a and Ch3 currents, and a double exponential function for 2a-N, 2b-TP, and 2a-T currents. First, the desensitization time constant of ASIC2a (1.06 ± 0.08 s, n = 7) was similar to the value previously reported by others^41^ (Fig. 4b). We then characterized the currents from Ch3 to determine whether Ch3 replicates the properties of ASIC2a. The desensitization time constant of Ch3 at pH 4.0 was 0.86 ± 0.05 s (n = 6) (Fig. 4b), demonstrating that desensitizing kinetics of Ch3 is not significantly different from that of ASIC2a. However, the peak current density of Ch3 at pH 3.5 was significantly smaller than that of ASIC2a (Fig. 4e), and pH-dependent response curve of Ch3 was shifted to the right compared with ASIC2a (Fig. 4f).

When the N-terminus in ASIC2a was replaced by that of ASIC2b (Fig. 3a, cf. 2a-N), desensitizing kinetics was considerably altered. At pH 4.0, the desensitization time constants of 2a-N currents were 0.30 ± 0.03 s (n = 6) for τ_1_ and 2.8 ± 0.1 (n = 6) for τ_2_ (Fig. 4c). In addition, 2a-N elicited readily desensitized currents. The cells expressing 2a-N produced only sustained currents to consecutive acid stimuli, and transient currents were eliminated; thus, we could not measure the pH-dependent peak current density in a single cell. Therefore, we plotted a pH-dependency curve by measuring the currents in individual cells. The peak current density of 2a-N was significantly decreased at pH 3.5 (Fig. 4e).

These alterations in biophysical properties of the currents were similarly observed when the N-terminus in Ch3 was exchanged to that of ASIC2b (Fig. 3a, cf. 2b-TP). At pH 4.0, the desensitization time constants of 2b-TP currents were 0.42 ± 0.03 s (n = 6) for τ_1_ and 5.1 ± 0.7 (n = 6) for τ_2_ (Fig. 4c), and 2b-TP also triggered desensitized currents to successive stimuli, suggesting a role of ASIC2a N-terminus in stabilizing the transient component of the currents. In addition, the peak current density of 2b-TP at pH 3.5 was significantly smaller than that of ASIC2a (Fig. 4e).

When the TM1 domain in ASIC2a was switched to that of ASIC2b (Fig. 3a, cf. 2a-T), desensitization time constants of the currents were 1.51 ± 0.20 s (n = 5) for τ_1_ and 5.2 ± 0.6 (n = 5) for τ_2_ (Fig. 4c). The pH-dependent peak current density of 2a-T reached a plateau near at pH 4.5, whereas that of ASIC2a did not reach the maximum current, even at pH 3.5 as previously reported^42^ (Fig. 4e). As shown in Fig. 4f, compared with ASIC2a, the relative currents of 2a-T significantly increased when the acidic stimuli (pH ≤ 5.0) were applied. However, the peak current density of 2a-T at pH 3.5 was significantly smaller than that of ASIC2a (Fig. 4e). Even though 2a-T has poor plasma membrane localization, it should be emphasized that we cannot rule out small fraction of 2a-T in the plasma membrane that can generate the currents. A half-maximal pH (pH_50_) value of 2a-T was 5.21 (Fig. 4f).

We also compared the ratio of sustained current to peak current (*I*_sus_/*I*_peak_) at pH 4.0. The ratio significantly increased in 2b-TP (0.15 ± 0.01, n = 9) compared to ASIC2a (0.07 ± 0.01, n = 7) (Fig. 4d). There was no statistically significant difference in Ch3 (0.06 ± 0.01, n = 10), 2a-N (0.09 ± 0.01, n = 8), or 2a-T (0.06 ± 0.01, n = 5) compared with ASIC2a. Taken all the data together, these results suggest that the N-terminus and the TM1 domain contribute to the gating properties of ASIC2 channels.

### H72 and E78 are critical for proton-sensitivity, whereas D77 is involved in determining subcellular localization and proton-sensitivity

**Figure 5:**
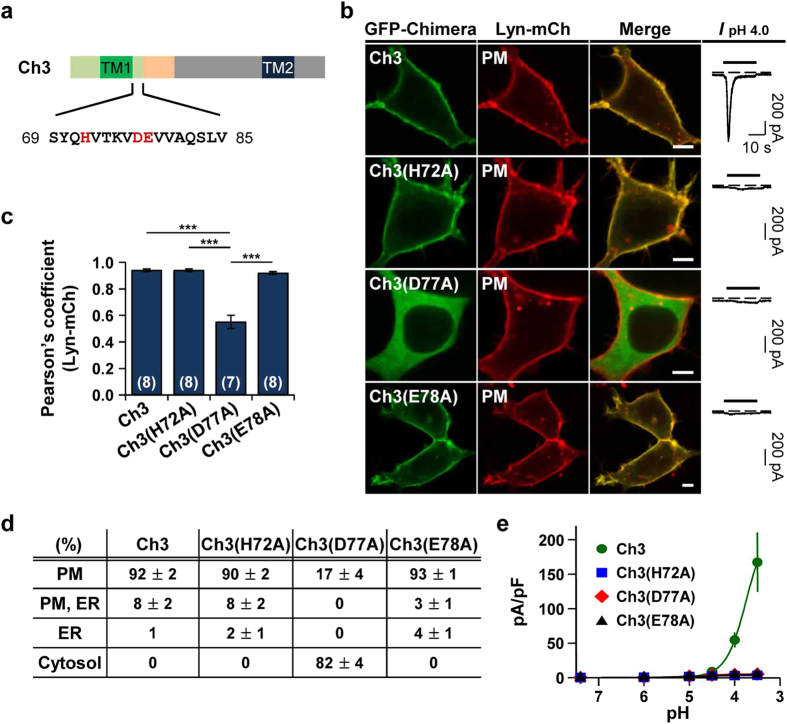
H72 and E78 are critical for proton-sensitivity, whereas D77 is involved in determining subcellular localization and proton-sensitivity. (**a**) Putative proton-binding sites (H72, D77, and E78) located in the proximal post-TM1 domain of Ch3 were highlighted in red. (**b**) Left, representative confocal images of HEK293T cells expressing each chimera with the plasma membrane marker, Lyn-mCh. The scale bar represents 5 μm. Right, pH 4.0-induced currents measured from the cells expressing each chimera. Dashed line indicates the zero current level. (**c**) Pearson’s correlation coefficient between the plasma membrane marker and each chimera (mean ± SEM, ***p < 0.001, with one-way ANOVA followed by Bonferroni post-hoc test). The number on each bar indicates n for each condition from three independent experiments. (**d**) Percentage of cells showing each chimera in specific subcellular localizations (mean ± SEM). For each chimera, 250 cells were counted from five independent experiments. (**e**) pH-dependent peak current density of each chimera (mean ± SEM). The time interval between pH applications is 2 min for a complete recovery from desensitization (Ch3, n = 5; Ch3(H72A), n = 5; Ch3(D77A), n = 6; Ch3(E78A), n = 5).

In the study with chimeras, we observed that the proximal post-TM1 domain of ASIC2a is required for generating proton-activated currents, even though the channels are expressed in the cell surface (Figs 2 and 3; Ch2, 2a-P, and 2b-T). This region contains histidine (H), aspartate (D), and glutamate (E), which are known to be putative proton-binding sites^19^,^20^ (Fig. 5a). To investigate the involvement of these residues in proton-sensitivity of Ch3, we made three single amino acid mutated channels (Ch3(H72A), Ch3(D77A), and Ch3(E78A)) and examined their subcellular distribution and proton-sensitivity. We observed that mutation at the position of H72 or E78 completely abolished proton-sensitivity, even though chimeric channels were still present at the cell surface (n = 5 for each) (Fig. 5b–e). These results indicate that H72 and E78 are critical residues for responding to extracellular protons, as previously reported^19^,^20^. However, we found that Ch3(D77A) lost not only surface expression but also proton-sensitivity (n = 6) (Fig. 5b–e). It suggests that D77 has a role in determining subcellular localization as well as proton-sensitivity of the channel.

### ASIC2b traffics to the cell surface by heteromeric assembly with ASIC2a

**Figure 6:**
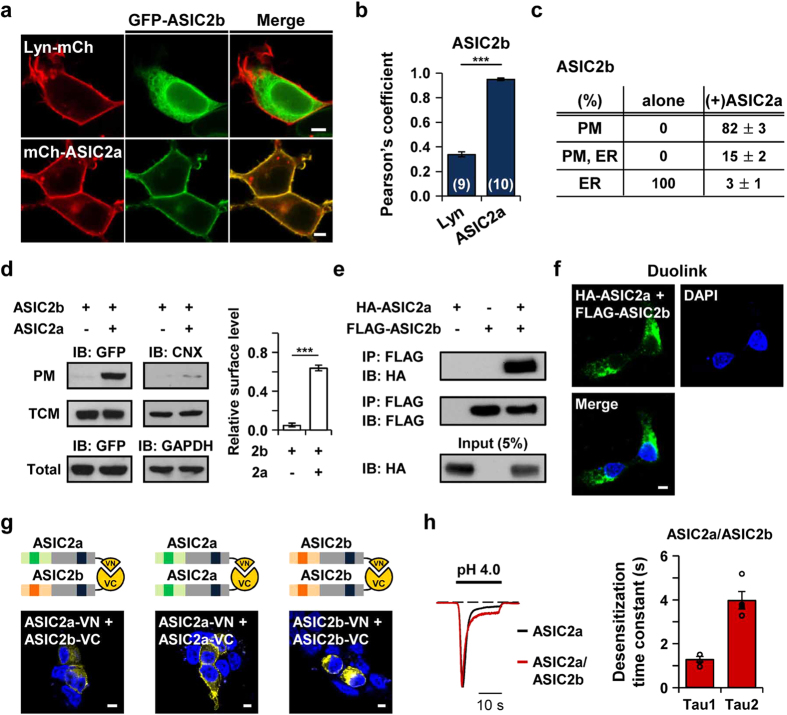
ASIC2b traffics to the cell surface by heteromeric assembly with ASIC2a. **(a)** Representative confocal images of HEK293T cells co-expressing ASIC2b with the plasma membrane marker (top) or ASIC2a (bottom). **(b)** Pearson’s correlation coefficient between ASIC2b and Lyn or ASIC2a (mean ± SEM, ***p < 0.001, with Student’s two-tailed unpaired *t*-test). The number on each bar indicates n for each condition from three independent experiments. **(c)** Percentage of cells showing ASIC2b in specific subcellular localizations in the absence or presence of ASIC2a (mean ± SEM). For each experiment, 250 cells were counted from five independent experiments. **(d)** Left, Western blotting on the plasma membrane (PM) fraction, total cellular membrane (TCM) fraction, and total lysate of cells expressing GFP-tagged ASIC2b with or without ASIC2a in pcDNA3.1(+) was performed using anti-GFP antibody. As controls, the PM and the TCM fractions were blotted using anti-calnexin (CNX) antibody, and total lysate was blotted using anti-GAPDH antibody. Right, the PM expression was normalized to the TCM expression (n = 3 for each, mean ± SEM, ***p < 0.001, with Student’s two-tailed unpaired *t*-test). **(e)** Co-immunoprecipitation assay in HEK293T cells co-expressing HA-ASIC2a and FLAG-ASIC2b. **(f)** Duolink PLA. Intense PLA signal was detected in HEK293T cells co-expressing HA-ASIC2a and FLAG-ASIC2b. **(g)** Schematic diagram of BiFC assay and confocal images of HEK293T cells expressing each combinatory construct. VN and VC are N- and C-terminal fragments of the Venus protein, respectively. Venus signals were examined for heteromerization of ASIC2a and ASIC2b, and homomerization of ASIC2a or ASIC2b. The fluorescent signals of BiFC were detected, as indicated by a yellow color. **(h)** Representative current traces of ASIC2a homomeric channel (black) and ASIC2a/ASIC2b heteromeric channel (red) were superimposed. Dashed line indicates the zero current level. Desensitization time constants (τ) of ASIC2a/ASIC2b current at pH 4.0 were calculated by fitting the current in a double exponential function (mean ± SEM). When Area1 and Area2 represent the area of τ_1_ and τ_2_, respectively, Area1/Area2 values were 1.39 ± 0.42 (n = 4). The scale bar represents 5 μm.

In physiological conditions, ASICs mostly exist as heteromeric channels^41^,^43^,^44^,^45^. According to the previous study, ASIC2a and ASIC2b are co-localized in a subpopulation of rat taste cells^13^. Based on this finding, we co-transfected fluorescent protein-tagged ASIC2a and ASIC2b in HEK293T cells. Strikingly, ASIC2b successfully targeted the cell surface when it was co-expressed with ASIC2a (Fig. 6a). In most of the cells, ASIC2b was co-localized with ASIC2a in the plasma membrane, as indicated by a high value of Pearson’s correlation coefficient between two subunits (Fig. 6b,c). Additionally, in the biochemical experiment, we observed that the relative surface level of ASIC2b was significantly increased in the presence of ASIC2a (Fig. 6d). To investigate the heteromeric interaction between two subunits in HEK293T cells, we performed co-immunoprecipitation (Co-IP) using HA-tagged ASIC2a and FLAG-tagged ASIC2b. The cell lysates were immunoprecipitated with an anti-FLAG antibody and then blotted with an anti-HA antibody. As shown in Fig. 6e, ASIC2a strongly associates with ASIC2b. We also examined the heteromeric assembly of ASIC2a and ASIC2b by using a Duolink proximity ligation assay (PLA) (Fig. 6f). Duolink PLA signals are generated when the distance between interacting proteins is <40 nm^46^,^47^. We detected a strong PLA signal from HA-ASIC2a and FLAG-ASIC2b in HEK293T cells (Fig. 6f).

The association between two subunits was further supported by a bimolecular fluorescence complementation (BiFC) assay, which allows visualization of subcellular localization of interacting proteins in living cells^48^ (Fig. 6g). To establish the Venus-based BiFC system, one complementary half of the Venus fluorescent protein, either the C-terminal fragment (VC) or the N-terminal fragment (VN), was fused to the C-terminus of ASIC2a or ASIC2b. The BiFC fluorescent signal was detected in the plasma membrane when ASIC2a-VN and ASIC2b-VC were co-transfected in HEK293T cells (Fig. 6g). As a positive control, ASIC2a homomers (ASIC2a-VN + ASIC2a-VC) showed a strong BiFC signal in the plasma membrane (Fig. 6g). For investigating whether ASIC2b also assembles by itself in the ER, we co-transfected ASIC2b-VN and ASIC2b-VC in HEK293T cells. Interestingly, we observed a strong BiFC signal in the ER, suggesting that ASIC2b has the ability to assemble by itself (Fig. 6g). As a negative control, we tested a BiFC signal between ASIC2b and unrelated ion channel that resides in the ER. We employed TWIK-1 or TREK-1, a member of two-pore domain K^+^ (K2P) channels. According to the previous study, TWIK-1/TREK-1 heterodimerization is required for cell surface expression of two subunits^47^. We detected no BiFC signals when ASIC2b-VN was co-expressed with TWIK-1-VC or TREK-1-VC in HEK293T cells, while co-expression of VN-TWIK-1 and TREK-1-VC showed a strong BiFC signal (Supplementary Fig. S4). No fluorescence was detected when ASIC2a, ASIC2b, TWIK-1, or TREK-1 with one half of Venus protein was expressed alone (Supplementary Fig. S4). Taken together, these results strongly suggest that ASIC2b requires an association with ASIC2a for its successful membrane trafficking.

We also examined whether ASIC2b can modulate the properties of ASIC2a currents at the cell surface. In heterologous expression systems, several electrophysiological studies have demonstrated that co-expression of ASIC2b alters the biophysical properties of ASIC channels, which has provided evidence for heteromeric association between ASIC2b and other subunits^13^,^17^,^44^. We also measured the currents from ASIC2a/ASIC2b heteromeric channels by co-expressing two subunits in HEK293T cells. Unlike the currents from ASIC2a homomeric channels, ASIC2a/ASIC2b currents desensitized with biphasic kinetics, as previously reported^13^ (Fig. 6h). We calculated the desensitization time constants for ASIC2a/ASIC2b currents using a double exponential function (1.29 ± 0.13 s (n = 4) for τ_1_ and 4.0 ± 0.4 (n = 4) for τ_2_ at pH 4.0) (Fig. 6h). The desensitization time constant for slow component of the currents was significantly increased in ASIC2a/ASIC2b currents compared to ASIC2a homomeric currents (***p < 0.001, with Student’s two-tailed unpaired *t*-test).

### Surface expression of ASIC2b is increased in the presence of ASIC2a in SH-SY5Y cells

**Figure 7:**
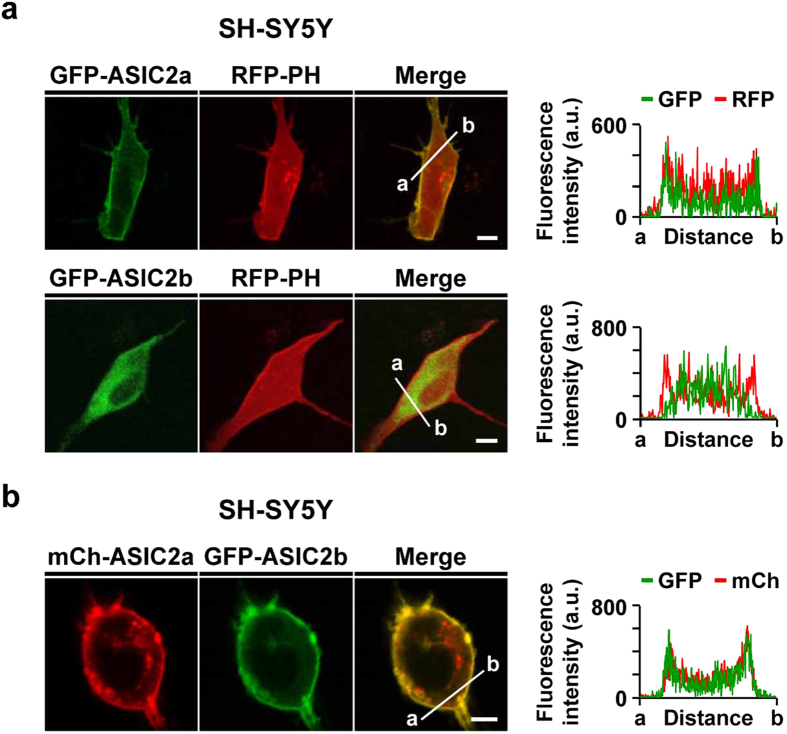
Surface expression of ASIC2b is increased in the presence of ASIC2a in SH-SY5Y cells. **(a)** Representative confocal images of SH-SY5Y cells expressing GFP-tagged ASIC2a or ASIC2b with the plasma membrane marker, RFP-PH. In SH-SY5Y cells, ASIC2a and ASIC2b are localized in the cell surface and the ER, respectively, as observed in HEK293T cells. Line scanning of fluorescent images was processed by using ZEN2011 software (Carl Zeiss). **(b)** Representative confocal images of SH-SY5Y cells co-expressing ASIC2a and ASIC2b. In the presence of ASIC2a, surface expression of ASIC2b was increased, as indicated by a yellow color in the merged image and line scanning. The scale bar represents 5 μm.

We also examined whether such membrane trafficking mechanisms of ASIC2 isoforms appear in SH-SY5Y cells, which are popularly used human neuroblastoma cells. GFP-tagged ASIC2a or ASIC2b was co-transfected with RFP-PH (PLCδ), which is a commonly used plasma membrane marker. Consistent with our observation in HEK293T cells, ASIC2a was predominantly localized in the cell surface like RFP-PH, as evidenced by overlapped line scanning results of GFP and RFP, while ASIC2b was accumulated in the ER in SH-SY5Y cells (Fig. 7a). However, when ASIC2b was co-expressed with ASIC2a, ASIC2b trafficked to the plasma membrane (Fig. 7b), as observed in HEK293T cells (Fig. 6).

### The N-terminal region of ASIC2a is necessary for the ASIC2a-dependent membrane targeting of ASIC2b

**Figure 8:**
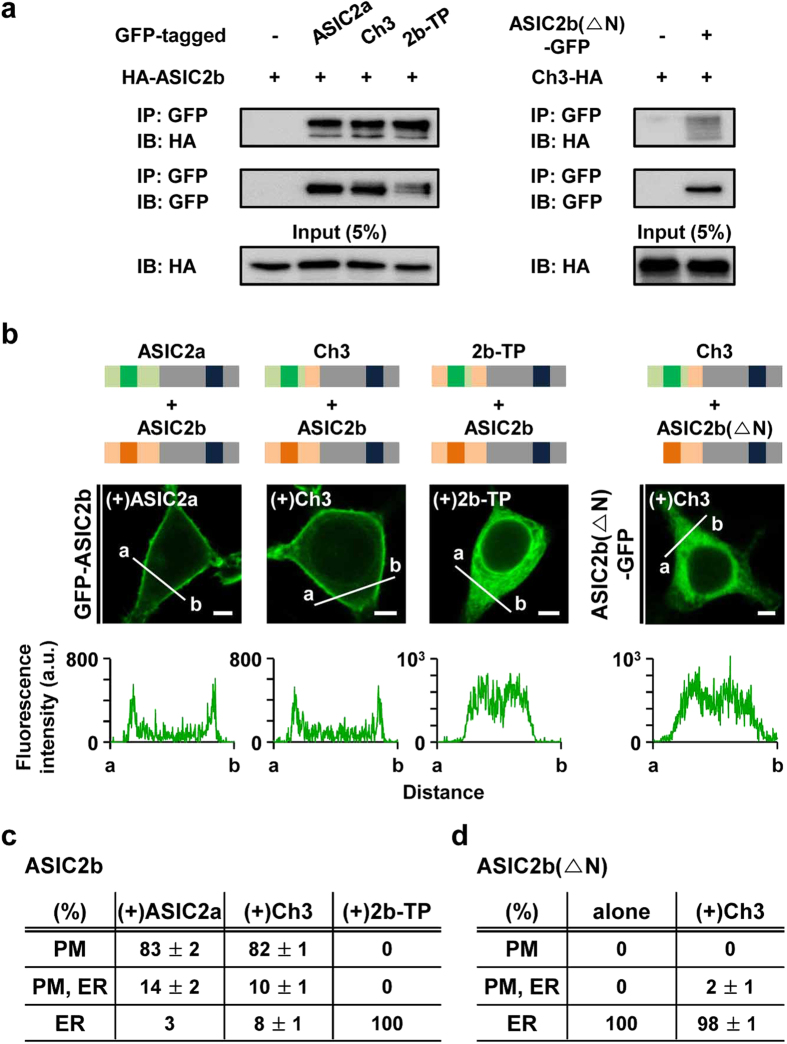
The N-terminal region of ASIC2a is necessary for the ASIC2a-dependent membrane targeting of ASIC2b. **(a)** Co-immunoprecipitation assay in HEK293T cells. GFP-tagged ASIC2 proteins and HA-ASIC2b (left) or GFP-tagged ASIC2b(△N) and Ch3-HA were co-transfected into HEK293T cells, and cell lysates were immunoprecipitated using anti-GFP antibody. The immunoprecipitates were then examined by Western blotting using anti-HA antibody. **(b)** Schematic diagram depicting heteromeric assembly experiments and representative confocal images of HEK293T cells expressing GFP-tagged ASIC2b in the presence of each ASIC2 protein in pcDNA3.1(+). Line scanning of fluorescent images was processed by using ZEN2011 software (Carl Zeiss). The scale bar represents 5 μm. **(c)** Percentage of cells showing ASIC2b in specific subcellular localizations in the presence of each ASIC2 protein in pcDNA3.1(+) (mean ± SEM). For each experiment, 250 cells were counted from five independent experiments. **(d)** Percentage of cells showing N-terminal deleted ASIC2b in specific subcellular localizations in the absence or presence of Ch3 (mean ± SEM). For each experiment, 250 cells were counted from five independent experiments.

Lastly, we tested whether Ch3 and 2b-TP containing the critical regions for membrane targeting can also deliver ASIC2b to the cell surface, like ASIC2a. First of all, we investigated heteromeric interaction between ASIC2b and Ch3 or 2b-TP by Co-IP experiments. As shown in Fig. 8a, both Ch3 and 2b-TP strongly associate with ASIC2b, like ASIC2a. However, when the N-terminal region of ASIC2b was deleted (ASIC2b(△N)), the interaction with Ch3 was markedly reduced (Fig. 8a). Based on these results, we investigated the subcellular distribution of GFP-tagged ASIC2b in the presence of Ch3 or 2b-TP. We observed that, like ASIC2a, Ch3 can also deliver ASIC2b to the cell surface, indicating that 100 amino acids (86 to 185) of ASIC2a extracellular domain are not considerably involved in surface targeting of Ch3/ASIC2b heteromeric channels (Fig. 8b,c). However, interestingly, ASIC2b remained in the ER when it was co-expressed with 2b-TP (Fig. 8b,c). It suggests that the N-terminal region from ASIC2a is required for membrane targeting of 2b-TP/ASIC2b heteromeric channels, although it does not seem to be considerably necessary for that of 2b-TP homomeric channels (Fig. 3). These results suggest that surface trafficking of homomers and heteromers could be differentially regulated.

When the N-terminal region of ASIC2b was deleted, this ASIC2b(△N) was also accumulated in the ER, and co-expression with Ch3 had no effect on its localization (Fig. 8b,d). It might be due to the reduced heteromeric interaction between two subunits, as observed in Co-IP experiments. Therefore, we can suggest that the N-terminus of ASIC2b is important for the heteromeric interaction with Ch3.

## Discussion

This study concerns different surface trafficking mechanism and proton-sensitivity of ASIC2 isoforms, ASIC2a and ASIC2b. We took advantage of the recombinant expression system to discern hidden trafficking mechanisms of ASIC2 proteins. We observed that two subunits display dramatically different subcellular localization when expressed alone in heterologous expression systems including HEK293T and neuroblastoma SH-SY5Y cells: ASIC2a targets the cell surface by itself, while ASIC2b resides in the ER. This finding was quite unexpected, because several studies previously reported that ASIC2b normally traffics to the cell surface^20^,^21^. However, quite recently, one research group reported that ASIC2a and ASIC2b have different subcellular distribution in NIH 3T3 cells^26^. In our study, we further investigated underlying mechanisms for this differential localization. By constructing a series of chimeras, we identified the TM1 and the proximal post-TM1 domain of ASIC2a as critical regions required for membrane targeting of ASIC2.

When these regions in ASIC2b were replaced by equivalent sequences of ASIC2a (Fig. 3, cf. 2b-TP), this chimera successfully trafficked to the cell surface. Moreover, this chimeric channel evoked proton-activated currents, although the biophysical properties were different from those of ASIC2a currents (Figs 3 and 4). The alterations in the current shape, time constant for desensitization, and desensitizing property to successive pH stimuli were largely dependent on the intracellular N-terminus. When the N-terminus in 2b-TP was replaced by that of ASIC2a (Fig. 2, cf. Ch3), this chimera produced ASIC2a-like currents in response to protons, although the current density was smaller than that of ASIC2a (Figs 2 and 4). Considering 2a-N and 2a-T, which showed similar current density to that of ASIC2a, we can infer that the decrease in the current density of Ch3 and 2b-TP might be due to the lack of ASIC2a amino acid sequences from 86 to 185, and that putative proton-binding sites involved in the current density might be present in this region.

In the chimera assay, we found that proton-sensitivity of Ch3, 2b-TP, 2a-N, and 2a-T are critically dependent on their proximal post-TM1 domain (17 amino acids) sequences from ASIC2a as well as surface expression. We could not detect any proton-activated currents from the chimeras containing the proximal post-TM1 domain sequences from ASIC2b, even though they were apparently expressed in the plasma membrane (Figs 2 and 3; Ch2, 2a-P, and 2b-T). Indeed, 17 amino acids from ASIC2a after the TM1 domain include H72, D77, and E78 of five putative proton-binding sites previously identified by others^19^,^20^. By using site-directed mutagenesis, we determined that H72 and E78 are critical residues for proton-sensitivity of Ch3 (Fig. 5). However, D77 seems to be involved in determining subcellular localization as well as proton-sensitivity of channels, inconsistent with the previous report^20^. Based on these results, we concluded that three amino acids (H72, D77, and E78) are all necessary for proton-sensitivity of channels. However, importantly, the chimera containing the proximal post-TM1 domain from ASIC2a was still insensitive to protons, since it was not expressed in the cell surface (Fig. 3, cf. 2b-P). This result indicates that the proximal post-TM1 domain from ASIC2a does not always ensure proton-sensitivity of channels.

Through these studies, we identified the minimum region of ASIC2a extracellular domain required for proton-sensing of ASIC2 proteins as the first 17 amino acids that include H72, D77, and E78 after the TM1 domain. These results are quite different with the previous study that identified the first 87 amino acids after the TM1 domain as the minimal region required for ASIC2 activation by protons^21^. In that study, they could not detect any proton-activated currents from five chimeras (AB3, AB4, AB10, AB11, and AB11-4). This was the case even though all of the chimeras contained H72, D77, and E78, which had been previously identified as putative proton-binding sites by the same group^20^. They reported that proton-insensitivity of these chimeras is due to inability of protons to activate the chimeric channels rather than disrupted surface expression^21^. Their study is different from ours in several points, such as species of clones, cell type used in the experiments, and the length of the TM1 domains. Our chimeras were constructed based on the sequences from the study of Jasti and colleagues (V43 to F68 for the TM1 of ASIC2a; A87 to L112 for the TM1 of ASIC2b)^15^, while they used shorter amino acid sequences for the TM1 domains. However, it is not clear whether these factors can account for the discrepancy in the minimal region of ASIC2a extracellular domain required for proton-sensitivity.

For the efficient trafficking of ASIC2 to the plasma membrane, orchestrated work of the TM1 domain and neighboring regions such as the N-terminus and the proximal post-TM1 domain is necessary. As illustrated in Fig. 3, exchange of the TM1 domain in ASIC2a to that of ASIC2b markedly decreased surface expression of the channel (cf. 2a-T). In addition, insertion of ASIC2a TM1 domain into ASIC2b promoted forward trafficking of ASIC2b (Fig. 3, cf. 2b-T), indicating that the TM1 domain of ASIC2a is critical for targeting the cell surface. However, when the N-terminus and the proximal post-TM1 domain were further replaced, the ability of the chimeric channel for trafficking was completely abolished (Fig. 3, cf. 2a-NTP) or enhanced (Fig. 2, cf. Ch3). These results indicate that neighboring regions of the TM1 domain are also involved in trafficking of the channel.

Finally, we showed that ASIC2b can traffic to the cell surface when it is co-expressed with ASIC2a. We verified the association between two subunits using Co-IP, Duolink PLA, and BiFC assay. According to the BiFC experiments, ER accumulation of ASIC2b is due to the lack of forward trafficking signals rather than inability to assemble by itself (Fig. 6). Surface trafficking of ASIC2b by the association with ASIC2a might be facilitated by membrane targeting domains that reside in ASIC2a. However, we also found that the N-terminal region of ASIC2a is necessary for the ASIC2a-dependent membrane targeting of ASIC2b (Fig. 8).

Recently, emerging role of ASIC2a in facilitating surface expression of ASIC1a has been reported^42^,^49^. Our finding further proves an important role of ASIC2a in surface trafficking of ASIC2b. We directly showed another critical meaning of heteromerization of ASICs other than the alterations in the biophysical properties of channels. However, we could not find a specific forward trafficking signal motif that might reside in the TM1 and the proximal post-TM1 domain of ASIC2a. Further investigation to find an anterograde signal that resides in ASIC2a and the role of ASIC2a in trafficking of other ASIC subunits will provide insight into surface targeting mechanisms of ASICs as well as strategies for developing therapeutic agents.

## Methods

### Cell culture and transfection

HEK293T and SH-SY5Y cells were obtained from Bertil Hille (University of Washington School of Medicine, Seattle, Washington) and Korean Cell Line Bank (Seoul National University, Seoul), respectively. Cells were cultured in DMEM supplemented with 10% FBS and 0.2% penicillin/streptomycin at 37 °C with 5% CO_2_, and plated in 35-mm culture dishes at 50–60% confluency a day before transfection. The cells were transiently transfected using Lipofectamine 2000 (Invitrogen) according to the manufacturer’s protocol. For the expression of homomeric ASICs, cells were transfected with 0.2 μg of various cDNAs. For the expression of heteromeric ASICs, we used each cDNA in a 1:1 molar ratio. The next day, transfected cells were plated onto poly-L-lysine (0.1 mg/ml, Sigma) coated chips. The fluorescent cells were studied 2 days after transfection.

### Plasmids

Mouse cDNA clones of ASIC2a (GenBank accession no. NM_001034013.2) and ASIC2b (GenBank accession no. NM_007384.3) were generously donated by Michael J. Welsh (University of Iowa, Iowa city, Iowa). The cDNAs encoding mouse ASIC2 subunits were amplified by PCR and cloned into pEGFP-C1 (Clontech), pEGFP-N1 (Clontech), and mCherry-C1 (Clontech) using EcoRI and KpnI sites, and pcDNA3.1(+) (Invitrogen) using HindIII and KpnI sites. The plasmids Lyn-mCherry and mCherry-Cb5 were kind gifts from Takanari Inoue (Johns Hopkins University School of Medicine, Baltimore, Maryland).

### Molecular cloning

For the generation of chimeras, we used the overlap extension PCR strategy^50^. The first PCRs were performed on two flanking regions using the primers containing the overlapping sequence of desired junction between ASIC2a and ASIC2b, and then the second PCR was performed using a mixture of the two PCR fragments from the first PCRs. The PCRs were carried out using the high-fidelity DNA polymerase, and products were subcloned into pEGFP-C1 (Clontech) and pcDNA3.1(+) (Invitrogen) for expression. Primers used for chimera construction are shown in Supplementary Table S1. For the single amino acid mutation and N-terminal deletion, we used a QuikChange Site-Directed Mutagenesis kit (Agilent). Primers used for mutagenesis are shown in Supplementary Table S2. All the chimeras and mutations were verified by DNA sequencing (Macrogen).

### Electrophysiology

Patch clamp recording using the whole-cell configuration was performed at room temperature (22–25 °C). Electrodes pulled from glass micropipette capillaries (Sutter Instrument) had resistances of 2–3 MΩ, and series resistance errors were compensated by >60%. Fast and slow capacitances were compensated before the application of test-pulse. We used a HEKA EPC-10 amplifier with pulse software (HEKA Elektronik) for recordings. For the fast perfusion of solution, we used a six channel perfusion valve control system (VC-6, Warner Instruments) with a quick change chamber narrow slotted bath (RC-46SNLP, Warner Instruments). Complete solution exchange is achieved within a second. The external solution used for recording the currents contained 160 mM NaCl, 5 mM KCl, 1 mM MgCl_2_, 2 mM CaCl_2_, and 10 mM HEPES, adjusted to pH 7.4 with tetramethylammonium hydroxide. For acidic solutions below pH 6.0, HEPES was replaced with MES. The pipette solution contained 140 mM KCl, 5 mM MgCl_2_, 10 mM HEPES, 0.1 mM 1,2-bis(2-aminophenoxy)ethane-*N,N,N’,N’*-tetraacetic acid (BAPTA), 3 mM Na_2_ATP, and 0.1 mM Na_3_GTP, adjusted to pH 7.4 with KOH. The currents were recorded by holding the cell at −70 mV. The pH pulses were applied every 2 min for a complete recovery from desensitization. The following reagents were obtained: BAPTA, Na_2_ATP, Na_3_GTP, and tetramethylammonium hydroxide (Sigma); HEPES (Calbiochem); MES (Alfa Aesar); and other chemicals (Merck). For the acquisition and analysis of data, we used Pulse/Pulse Fit software in combination with an EPC-10 patch clamp amplifier (HEKA Elektronik) and Igor Pro (WaveMetrics, Inc.). The pH-dependency curve was fitted with a Hill equation. For measuring the desensitization time constant, a single or double exponential function was used. When a double exponential function was used, relative areas of time constants were calculated by integrating the functions, y_1_ = *A*_1_exp{− (x − x_0_)/τ_1_} and y_2_ = *A*_2_exp{− (x − x_0_)/τ_2_} (*A*, coefficient; x, time; τ, tau). Further data processing was performed with Excel 2012 (Microsoft) and Igor Pro (WaveMetrics, Inc.).

### Plasma membrane fraction and Western blotting

Plasma membrane fraction was isolated using a Plasma Membrane Protein Extraction kit (abcam, ab65400) following the manufacturer’s instructions. In brief, cells were lysed by homogenize buffer with a protease inhibitor cocktail. Homogenates were centrifuged at 700 × *g* for 10 min at 4 °C. Pellets containing plasma membrane and organelle membranes were isolated from the cytosol fraction by high-speed centrifugation of the supernatants at 10,000 × *g* for 30 min at 4 °C. To isolate the plasma membrane fraction further, pellets were re-suspended in Upper Phase buffer and were extracted in Lower Phase buffer. This was followed by centrifugation to pellet the plasma membrane fraction. Plasma membrane pellets were solubilized with 0.5% Triton X-100 in PBS for Western blotting.

For immunoblotting, protein samples were separated by SDS-PAGE using 8–10% gels. The separated proteins were transferred to polyvinylidene fluoride membranes, and blotted with anti-GFP (ThermoFisher Scientific; 4B10B2, 1:2,000), anti-calnexin (Enzo; ADI-SPA-860, 1:2,000), anti-GAPDH (Cell Signaling; #2118, 1:10,000), or anti-HA (Bethyl; A190-108A, 1:1,000) antibodies. After washing blots, proteins were visualized using an ECL detection system (Bio-Rad). Quantitative analysis was performed with ImageJ.

### Co-Immunoprecipitation (Co-IP) and Western blotting

HEK293T cells were lysed with HEPES buffer (20 mM HEPES, pH 7.5, 150 mM NaCl, 1% NP-40, 0.1% SDS, and 1 mM NaF) containing a protease inhibitor cocktail. Whole-cell lysates were incubated on ice for 30 min and then cleared at 13,000 rpm for 30 min at 4 °C. For Co-IP, the supernatants were incubated overnight at 4 °C with anti-FLAG (Sigma; M2) or anti-GFP (ThermoFisher Scientific; 4B10B2, 1:2,000) antibodies, followed by incubation with protein A/G PLUS-agarose beads for 1 h. For immunoblotting, protein samples were separated by SDS-PAGE using 10% gels. The separated proteins were transferred to polyvinylidene fluoride membranes. The blots were incubated overnight at 4 °C with anti-HA (Roche Applied Science; 3F10, 1:1,000), anti-FLAG (Sigma; F1804, 1:1,000), or anti-GFP (ThermoFisher Scientific; 4B10B2, 1:2,000) antibodies. After washing blots, they were incubated with horseradish peroxidase-conjugated secondary antibody for 1 h at room temperature and visualized using an ECL detection system (Bio-Rad).

### BiFC assay

For BiFC, ASIC2a and ASIC2b were cloned into bimolecular fluorescence complement (pBiFC)-VN173 and pBiFC-VC155 vectors. HEK293T cells were co-transfected with cloned BiFC vectors in all possible pairwise combinations. After 24 h, these cells were fixed with 4% paraformaldehyde for 20 min at room temperature and mounted with Dako Fluorescence Mounting Medium. Venus fluorescence signals were observed with an Olympus Fluoview FV1000 confocal microscope (Olympus) at room temperature.

### Duolink proximity ligation assay

Interaction was detected by a Duolink Proximity Ligation Assay kit (Olink Bioscience, Uppsala, Sweden: PLA Probe anti-Mouse MINUS; PLA Probe anti-Rabbit PLUS; Detection Kit 563). The PLA Probe anti-Mouse MINUS binds to the HA antibody (Cell Signaling; #2367), whereas the PLA Probe anti-Rabbit PLUS binds to the FLAG antibody (Cell Signaling; #2368). After preincubation with a blocking agent for 1 h, the fixed HEK293T cells were incubated overnight with the primary antibodies to anti-HA (Cell Signaling, 1:100) and anti-FLAG (Cell Signaling, 1:100). Duolink PLA probes detecting mouse or rabbit antibodies were diluted in the blocking agent to a concentration of 1:5 and applied to the slides, followed by incubation for 1 h in a pre-heated humidity chamber at 37 °C. Unbound PLA probes were removed by washing. The slides were then incubated in a ligation solution consisting of Duolink Ligation stock (1:5) and Duolink Ligase (1:40) for 30 min at 37 °C. Detection of the amplified probe was performed with the Duolink Detection kit. Duolink Detection stock was diluted at 1:5 and applied for 100 min at 37 °C. Final washing steps were carried out in saline sodium citrate buffer. Duolink PLA signals were observed with an Olympus Fluoview FV1000 confocal microscope (Olympus) at room temperature.

### Confocal imaging

The living cells were imaged 2 days after transfection on poly-L-lysine coated chips with a Carl Zeiss LSM 700 confocal microscope (Carl Zeiss) at room temperature. The external solution contained 160 mM NaCl, 5 mM KCl, 1 mM MgCl_2_, 2 mM CaCl_2_, and 10 mM HEPES, adjusted to pH 7.4 with tetramethylammonium hydroxide. Images were scanned with a 40× (water) objective lens at 1024 × 1024 pixels using a digital zoom. All confocal images were transferred from LSM5 to TIFF format.

### Quantitative analysis of fluorescent images

Quantitative analysis of confocal images was carried out using ZEN2011 software (Carl Zeiss) and ImageJ. Pearson’s correlation coefficients of multiple sets of images were quantified by the ‘Colocalization’ tool in the ImageJ. The values are between 0 and 1; a value of 1 means complete co-localization, while a value of 0 means no co-localization. Line scanning of fluorescent images was processed by using the ‘Profile’ tool in ZEN2011 software (Carl Zeiss).

To determine the percentages of cells showing each construct in specific subcellular localizations, we manually counted cells co-transfected with a specific organelle probe. For each condition, we counted 250 cells from five independent experiments. The raw data were processed with Excel 2012 (Microsoft) and Igor Pro (WaveMetrics, Inc.).

### Statistical analysis

All quantitative data are represented as mean ± SEM. Comparisons between two groups were analyzed using Student’s two-tailed unpaired *t*-test. The significance of data among more than two groups was assessed by one-way ANOVA followed by Bonferroni post-hoc test. Differences were considered significant at the * p < 0.05, **p < 0.01, and ***p < 0.001 levels, as appropriate.

## Additional Information

**How to cite this article**: Kweon, H.-J. *et al*. Acid-Sensing Ion Channel 2a (ASIC2a) Promotes Surface Trafficking of ASIC2b via Heteromeric Assembly. *Sci. Rep.* **6**, 30684; doi: 10.1038/srep30684 (2016).

## Supplementary Material

Supplementary Information
