# Supplementary material for: Acid-Sensing Ion Channel 2a (ASIC2a) Promotes Surface Trafficking of ASIC2b via Heteromeric Assembly
Source: Sci Rep. 2016 Aug 1;6:30684. doi: 10.1038/srep30684 (PMC4967927; doi:10.1038/srep30684)
Supplement: Supplementary Information [file srep30684-s1.pdf]

Supplementary Information

Acid-Sensing Ion Channel 2a (ASIC2a) Promotes Surface Trafficking of ASIC2b via Heteromeric Assembly

Hae-Jin Kweon[1], Dong-Il Kim[1], Yeonju Bae[2], Jae-Yong Park[2], and Byung-Chang Suh[1]

[1]Department of Brain & Cognitive Sciences, DGIST, Daegu 42988, Republic of Korea

[2]School of Biosystem and Biomedical Science, College of Health Science, Korea University, Seoul 02841, Republic of Korea

Supplementary Tables

Table S1. Primers and templates used for chimera construction.

	Fragment 1		Fragment 2		Template	
	FP (5’-3’)	RP (5’-3’)	FP (5’-3’)	RP (5’-3’)	Fragment 1	Fragment 2
**Ch1**	tcgaattctatggacctcaaggagagc	ggccagcacccacagcgcacgccggatggtcagcgg	ccgctgaccatccggcgtgcgctgtgggtgctggcc	gcggtacctcagcaggcaatctcctc	ASIC2a	ASIC2b
**Ch2**	tcgaattctatggacctcaaggagagc	tcgcgtgtgtgacgggaatgagaaatagtaggatac	gtatcctactatttctcattcccgtcacacacgcga	gcggtacctcagcaggcaatctcctc	ASIC2a	ASIC2b
**Ch3**	tcgaattctatggacctcaaggagagc	ggggttgttgttgcacacggtcacagctgggaagac	gtcttcccagctgtgaccgtgtgcaacaacaacccc	gcggtacctcagcaggcaatctcctc	ASIC2a	ASIC2b
**2a-P**	tcgaattctatggacctcaaggagagc	gagggtcacagctgggaacggcagctggcggctcca	tggagccgccagctgccgttcccagctgtgaccctc	gcggtacctcagcaggcaatctcctc	Ch2	ASIC2a
**2a-T**	tcgaattctatggacctcaaggagagc	ggtaacatgctgatatgagagccagtagagcaggcg	cgcctgctctactggctctcatatcagcatgttacc	gcggtacctcagcaggcaatctcctc	Ch1	ASIC2a
**2a-N**	tcgaattctatgagccggagcggcggagcccggctg	ggccactgcccaaagcacccgccgctggaaagagcc	ggctctttccagcggcgggtgctttgggcagtggcc	gcggtacctcagcaggcaatctcctc	ASIC2b	ASIC2a
**2a-NTP**	tcgaattctatgagccggagcggcggagcccggctg	gccattgaggttgcagagggtgacagcggggaacgg	ccgttccccgctgtcaccctctgcaacctcaatggc	gcggtacctcagcaggcaatctcctc	ASIC2b	ASIC2a
**2b-P**	tcgaattctatgagccggagcggcggagcccggctg	ggtaacatgctgatatgagagccagtagagcaggcg	cgcctgctctactggctctcatatcagcatgttacc	gcggtacctcagcaggcaatctcctc	ASIC2b	Ch3
**2b-T**	tcgaattctatgagccggagcggcggagcccggctg	cgtgtgtgacgggaaactgaaatagtaggatactct	agagtatcctactatttcagtttcccgtcacacacg	gcggtacctcagcaggcaatctcctc	2b-TP	ASIC2b
**2b-TP**	tcgaattctatgagccggagcggcggagcccggctg	ggccactgcccaaagcacccgccgctggaaagagcc	ggctctttccagcggcgggtgctttgggcagtggcc	gcggtacctcagcaggcaatctcctc	ASIC2b	Ch3

Table S2. Primers used for mutagenesis.

	**Sense (5'-3')**	**Antisense (5'-3')**
**Ch3(H72A)**	atcctactatttctcatatcaggctgttaccaaggtggatgaagtg	cacttcatccaccttggtaacagcctgatatgagaaatagtaggat
**Ch3(D77A)**	catgttaccaaggtggctgaagtggtggcccag	ctgggccaccacttcagccaccttggtaacatg
**Ch3(E78A)**	ttaccaaggtggatgcagtggtggcccagag	ctctgggccaccactgcatccaccttggtaa
**ASIC2b(△N)**	gcgctgtgggtgctggccttc	catgaattcgaagcttgagctcga

Supplementary Figures

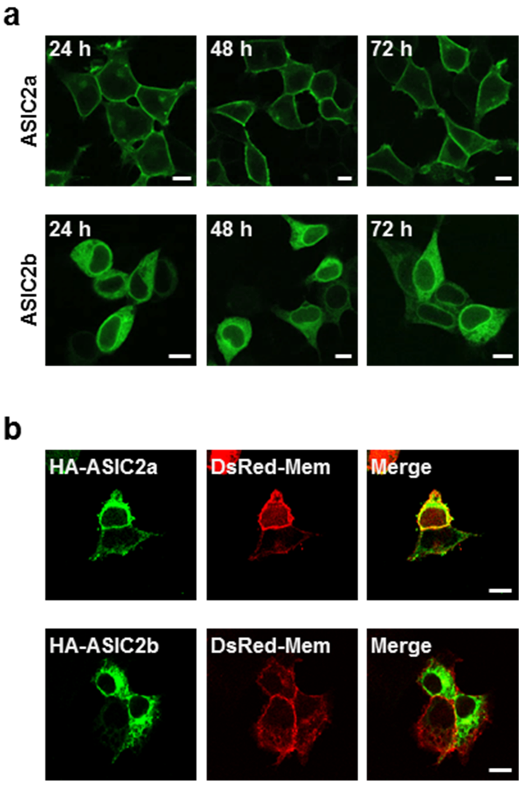

Figure S1. Differential subcellular localization of ASIC2a and ASIC2b. (a) Subcellular localization of GFP-tagged ASIC2a or ASIC2b was examined after different days of transfection. ASIC2a is localized in the plasma membrane already 1 day after transfection, while ASIC2b remained in the ER even 3 days after transfection. **(b)** Immunocytochemistry on HEK293T cells co-expressing the plasma membrane marker DsRed-Mem and HA-ASIC2a (top) or HA-ASIC2b (bottom) by anti-HA antibody. The scale bar represents 10 μm.

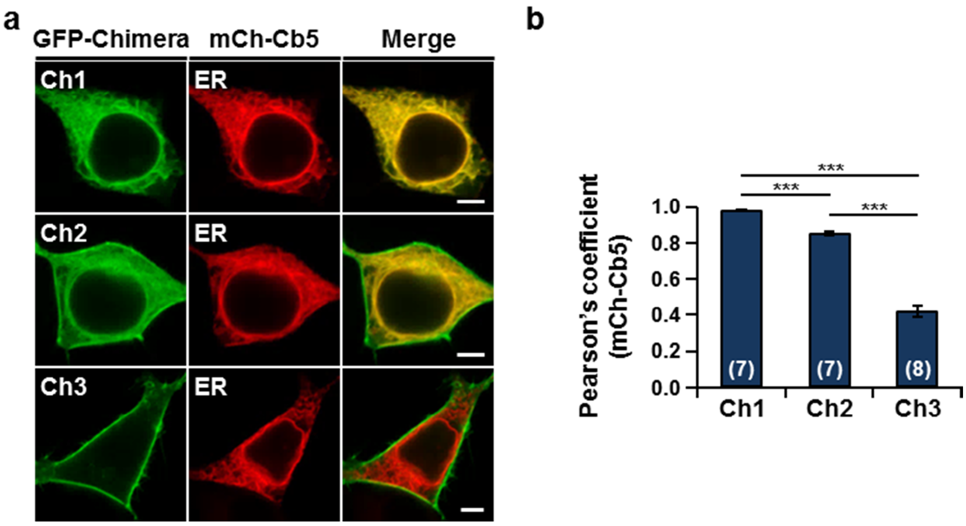

Figure S2. Chimera assay with an ER marker. (a) Representative confocal images of HEK293T cells expressing each chimera with the ER marker, mCh-Cb5. The scale bar represents 5 µm. **(b)** Pearson's correlation coefficient between the ER marker and each chimera (mean ± SEM, *** $p < 0.001$, with one-way ANOVA followed by Bonferroni post-hoc test). The number on each bar indicates n for each condition from three independent experiments.

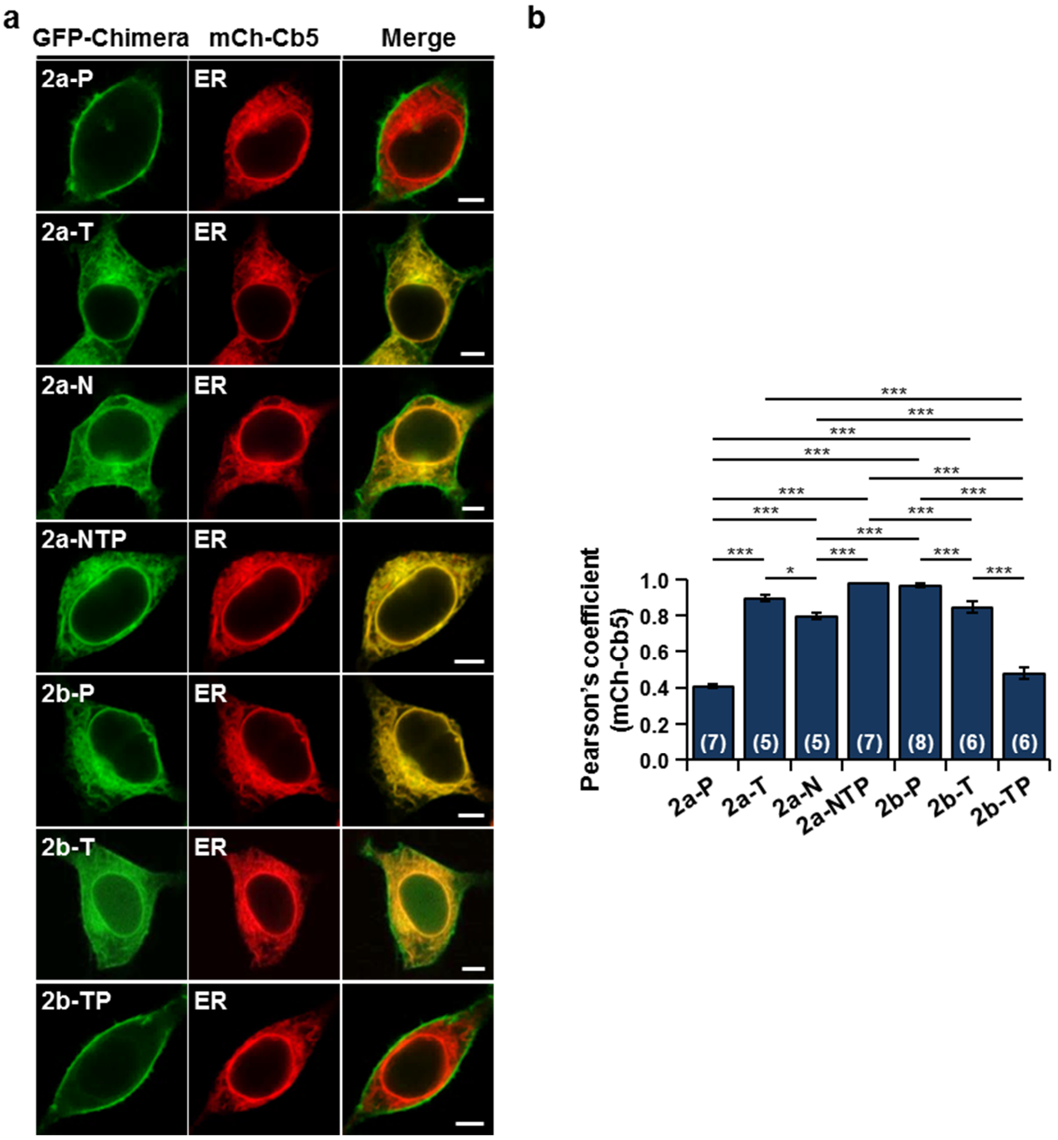

Figure S3. Chimera assay with an ER marker. (a) Representative confocal images of HEK293T cells expressing each chimera with the ER marker mCh-Cb5. The scale bar represents 5 µm. **(b)** Pearson's correlation coefficient between the ER marker and each chimera (mean ± SEM, * $p < 0.05$, *** $p < 0.001$, with one-way ANOVA followed by Bonferroni post-hoc test). The number on each bar indicates n for each condition from three independent experiments.

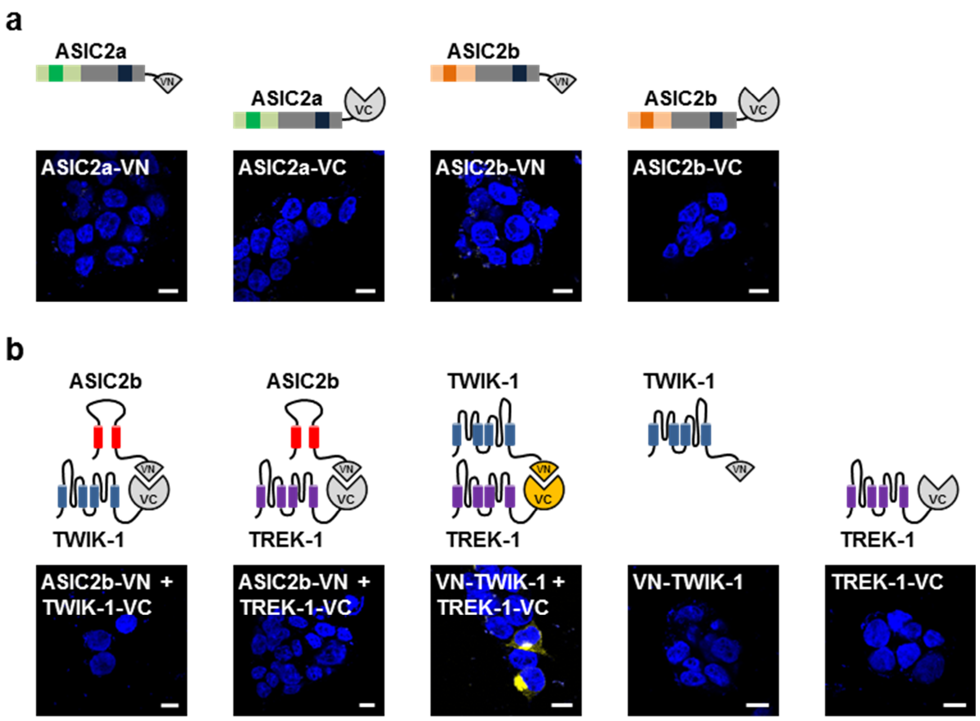

Figure S4. Control experiments for BiFC assay. (a) For determination of specificity of BiFC, HEK293T cells were transfected with one half of split Venus protein. No fluorescence was detected when either ASIC2a or ASIC2b with one half of Venus protein was expressed alone. **(b)** ASIC2b-VN was tested with TWIK-1-VC or TREK-1-VC. BiFC signal was detected in cells co-expressing VN-TWIK-1 and TREK-1-VC. No fluorescence was detected when either TWIK-1 or TREK-1 with one half of Venus protein was expressed alone. The scale bar represents 10 µm.

Supplementary Methods

Immunocytochemistry

HEK293T cells growing on coverslips were co-transfected with HA-tagged vectors and Ds-red membrane marker using PEI (Sigma). The cells were fixed in 4% paraformaldehyde for 20 min at room temperature, and then permeabilized with PBS with 0.1% Triton-X100 for 3 min. Non-specific binding was prevented with 1 h incubation in 5% BSA (Bovogen). Cells were incubated overnight, at 4°C, with anti-HA antibody (Roche Applied Science; 3F10, 1:200). After washing, DyLight 488-conjugated secondary antibody (Jackson Labs, 1:400) was added and incubated for 1 h at room temperature. The cells were washed and mounted, and then observed with an Olympus Fluoview FV1000 confocal microscope (Olympus).
